# The food and beverage cues in digital marketing model: special considerations of social media, gaming, and livestreaming environments for food marketing and eating behavior research

**DOI:** 10.3389/fnut.2023.1325265

**Published:** 2024-02-06

**Authors:** Sara J. Maksi, Kathleen L. Keller, Frank Dardis, Martina Vecchi, Jason Freeman, Rebecca K. Evans, Emma Boyland, Travis D. Masterson

**Affiliations:** ^1^Department of Nutritional Sciences, The Pennsylvania State University, University Park, PA, United States; ^2^Department of Advertising and Public Relations, The Pennsylvania State University, University Park, PA, United States; ^3^Department of Agricultural Economics, Sociology and Education, The Pennsylvania State University, University Park, PA, United States; ^4^Department of Advertising, School of Communications, Brigham Young University, Provo, UT, United States; ^5^Department of Psychology, The University of Liverpool, Liverpool, United Kingdom

**Keywords:** food marketing, digital media, eating behavior, social media, children and adolescent, health, policy

## Abstract

Digital marketing to children, teens, and adults contributes to substantial exposure to cues and persuasive messages that drive the overconsumption of energy dense foods and sugary beverages. Previous food marketing research has focused on traditional media, but less is known about how marketing techniques translate within digital platforms, such as social media, livestreaming, and gaming. Building upon previous theories and models, we propose a new model entitled food and beverage cues in digital marketing (FBCDM). The FBCDM model specifies key marking elements and marketing integration strategies that are common on digital platforms and are hypothesized to enhance the effects of advertising and incentive sensitization process. FBCDM also categorizes measurable outcomes into three domains that include brand, food, and social outcomes. Additionally, repeated marketing exposure and the resulting outcomes are hypothesized to have long term consequences related to consumer markets, consumption behavior, culture, and health. We include a discussion of what is currently known about digital marketing exposure within the outcome domains, and we highlight gaps in research including the long-term consequences of digital marketing exposure. The FBCDM model provides a conceptual framework to guide future research to examine the digital marketing of food and beverages to children and adolescents in order to inform government and industry policies that restrict the aggressive marketing of products associated with obesity and adverse diet related outcomes.

## Introduction

Food and beverage marketing is a major contributor for establishing a preference and drive to eat energy-dense, nutrient-poor foods (1–3). Concern regarding exposure to food and beverage marketing and rising obesity rates has prompted policies to restrict advertising to children in some countries (4). Additionally, the World Health Organization has included the need to reduce exposure to this marketing as one of their specific aims to address the childhood obesity epidemic and encourages member states to use policies and legislation to achieve this goal (5, 6). However, most policies attempting to restrict advertising to youth have primarily focused on television and do not adequately or directly address current technology and digital media (4). Popular digital platforms, such as Facebook, Instagram, Twitch, and Tik Tok, have monthly active users in the billions and have primarily adolescent and young adult audiences (7–10). Only recently has digital media been included in regulation discussions (11, 12). Food and beverage marketing on digital media is especially concerning for children and adolescents given the broad range of digital exposures (11, 13).

A wide variety of data driven marketing strategies on digital media platforms are being used by food and beverage brands to market directly to consumers in a manner that encourages consumption of energy drinks, sugary beverages, snack foods, and fast-food meals, which are linked with poor diet quality and adverse health outcomes when consumed in excess or in place of more nutrient dense foods (14, 15). These strategies target children and adolescents through the use of emotional appeals, social influence, and interactive features. In a qualitative study, adolescents reported that food marketing contributed to their craving for and purchasing of specific brands, inducing emotional responses and prompting engagement with branded content or social media (16). However, digital platforms (Table 1) evolve rapidly with advances in technology that can make understanding their impact on health difficult to track and regulate.

One emerging media format on digital platforms – livestreaming – provides a clear illustration for the complexity of digital media and the need for additional research on the effects of food marketing within these spaces. Livestreaming involves synchronous online broadcast by content creators, colloquially termed “streamers,” who have the ability to interact with their audience in real time. Streamers are similar to influencers on other social media platforms as they use their social capital to build financially lucrative partnerships with brands and promote those brands through their livestreams and on their social media pages. Influencer marketing across platforms is estimated to grow to a worth of $21 billion in 2023 with $5.20 return on investment for every dollar spent (17). Livestreaming originates from the online gaming community, but this medium has branched out rapidly into other content areas such as music, cooking, and beauty (18). The most popular video game livestreaming platform (19, 20), Twitch, has 33.2 million users in the U.S alone, with billions of hours watched per year (21). In 2020, adolescents and young adults accounted for 37.8 and 40.6% of the total users, respectively (22). To reach this young audience, food brands are investing marketing efforts into not only the livestream platforms, but also with popular streamers and esports leagues that competitively play video games (19, 23).

The foundational research of marketing techniques employed in traditional media (TV, movies, and print) offers a starting point at which to examine the possible implications of food marketing within digital platforms. Previous work on the effects of food marketing on eating behaviors has led to the development of a general explanatory model entitled the Reactivity to Embedded Food Cues in Advertising Model (REFCAM) (24). This model has been used previously to describe how food marketing is likely to influence and reinforce the consumption of advertised foods (24). In this model, food marketing is hypothesized to induce physiological and psychological processes that drive the viewer to seek out and consume the advertised foods, and this consumption in turn increases the individual’s susceptibility to future encounters with food marketing (24). Another model, the Hierarchy of Unhealthy Food Promotion Effects, provides greater description of the multitude of marketing effects (25). This model theorizes that direct effects on intake occur downstream of brand awareness, brand attitudes, and purchase intentions. The unique aspects of digital media platforms that influence marketing strategies and exposure are further described in the How Digital Marketers Target Youth framework. This framework presents the idea that digital marketing transcends platforms specific messaging, allows for dynamic engagement that includes user generated content, and leverages social influence to create particularly salient persuasive messaging (26). While these models provide broad utility to researchers, there is a need for a more specialized model to better account for the unique aspects of food and beverage presentation and medium integration that are now possible within digital media, and which set it apart from other media in terms of how it is experienced by young people.

**Table 1:** Definition of key terms.

*Digital media*	Media that is disseminated electronically or online.
*Digital food and beverage marketing*	Marketing that occurs on or within digital media.
*Social media*	Websites or apps whose primary purpose is to share content and information among peer networks.
*Livestreaming*	Content shared synchronously with real time response from viewers through a chat box. A feature on social media platforms, Facebook, Instagram, and YouTube. Dedicated livestreaming platforms have primarily focused on video-game content.
*Streamer*	The content creator on livestreaming platforms. Most commonly refers to video-game livestreaming where the streamer plays a video game while interacting with the viewers.
*Influencer marketing*	Partnerships or paid promotions between brand and an influencer, typically on social media platforms. This marketing involves brand placement, discussion, and modelled use by the influencer.
*Influencer*	An individual with a large following on a digital platform, typically a social media platform, that leverages their following to earn income to promote brands and products.
*User-generated content*	Content created typically on social media platforms that features a brand or product but is not monetarily compensated and messaging is not determined by the brand.

**Figure 1:**
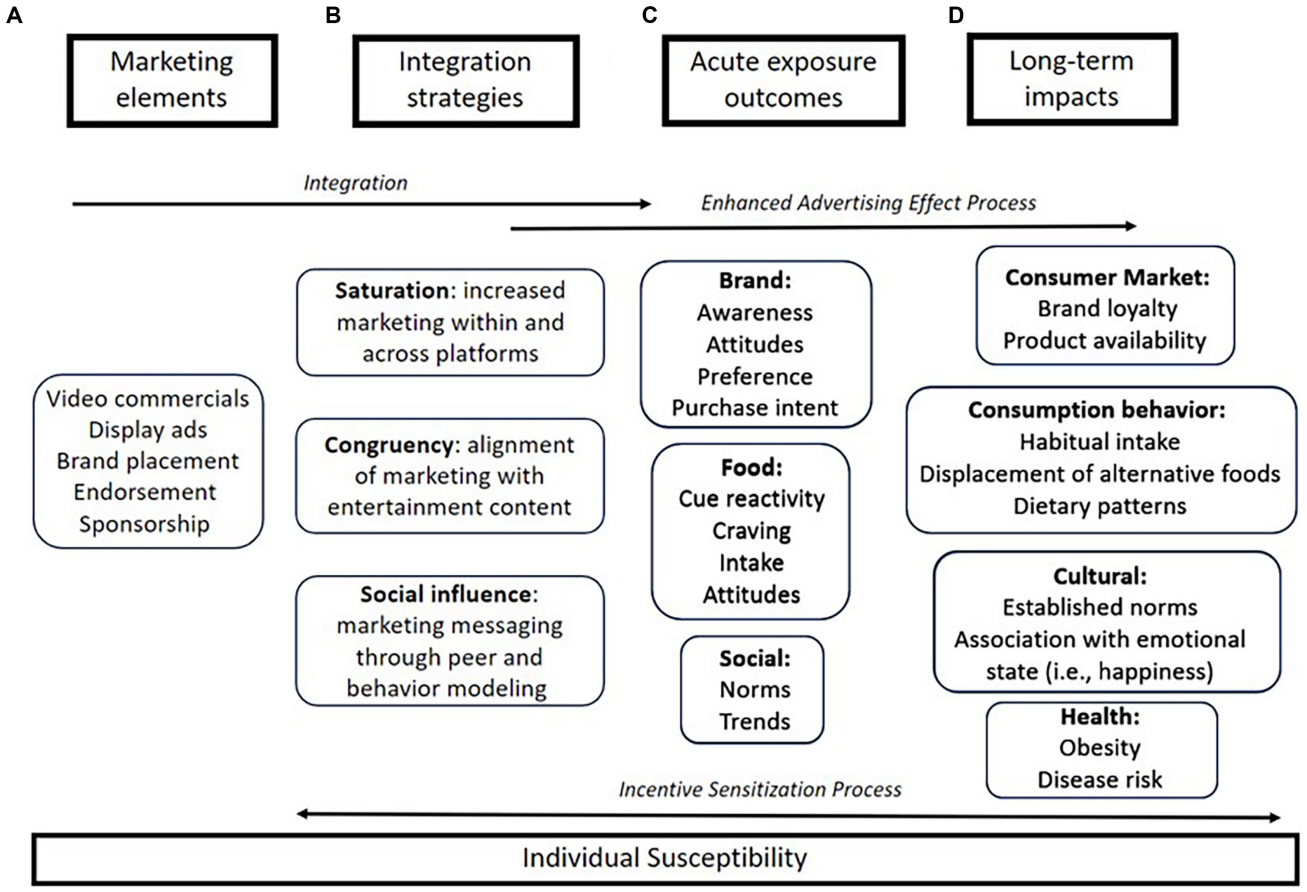
The food and beverage cues in digital marketing (FBCDM) model. **(A)** Marketing elements are defined here as the types of individual advertisements that are used within digital media. **(B)** The marketing elements are integrated into digital media in a variety of ways that may enhance the advertising effect process. These strategies include increased saturation, improved congruency, and powerful social influence. **(C)** Acute exposure outcomes can be categorized by brand specific, intake, and social based effects. **(D)** Repeated exposure and incentive sensitization process that reinforces the effects of marketing can lead to long term impacts on consumption behaviors, purchasing, and health.

Accordingly, we propose to build upon and merge these models and their underlying theories with a revised model, entitled the food and beverage cues in digital marketing (FBCDM) model. This model highlights the various types of marketing elements (e.g., ads, endorsement) that can be present simultaneously and considers various levels of marketing integration (e.g., saturation, congruency, and social influence), that are possible on digital platforms (see Figure 1). Additionally, elements from the Hierarchy of Unhealthy Food Promotion Effects model are incorporated to acknowledge the multiple points of impact that food marketing may influence prior to food consumption (25). The effects of digital marketing are categorized into measurable outcomes that fall into three domains (brand, food, and social) and long-term impacts that span consumer behavior, consumption behavior, cultural norms, and health. This model also hypothesizes that individual susceptibility will alter how marketing is perceived and the resulting impacts of exposure. This conceptual model aims to provide a framework to inform future hypothesis testing that addresses the complexities and power of food marketing within digital multimedia. In the discussion that follows, we will review key components of the proposed FBCDM model with the aim of understanding their impact on consumer attitudes and behavior. To illustrate how these concepts are currently presenting within digital media, the livestreaming platform Twitch will serve as the baseline example, but we propose that these concepts are applicable to other digital outlets such as video and social media platforms.

## Online marketing elements

Online platforms utilize a variety of marketing elements to convey their messages, including static advertising, video advertising, product placement, and endorsement (Table 2). In this section, we describe each of these marketing elements highlighted in the FBCDM model, with a particular emphasis on what is known in relation to food marketing.

**Table 2:** Description and examples of marketing elements.

Marketing elements	Description	Examples	
		*Traditional media*	*Digital media*
*Video commercials*	A short, 15-30 second, video featuring branded imagery, music, and storyline to invoke an impression on the viewer.	Television commercial breaks during a program	Before, during, or after viewing video content (YouTube, Twitch)
*Display ads*	A static image of a brand logo, product image, and/or slogan.	Print (magazines) and billboards	Present in multiple locations on websites and apps. Placed on top of entertainment content (Twitch, YouTube) or in feeds (Facebook, Instagram, Tik Tok)
*Brand placement*	Brand logo or product placed into entertainment content. The placement can be interacted with or in the background.	Characters in television shows and movies use a product in a scene or it is placed in the background.	Brand logos or products are present within an image or video content that is not explicitly promoted or acknowledged as a paid advertisement.
*Endorsement*	A product or brand is promoted by a trusted or popular source (celebrity, athlete, musician).	Endorsement used in print advertisements and video commercials. Depicts the endorser holding or consuming the product.	Can be present in display ads or commercial on digital platforms. Takes the form of influencer marketing on social media and traditional celebrities also endorse products on their own social media accounts in addition to brand owned advertisements.
*Sponsorship*	A brand provides money towards an event with the return of having their branded material present during the event.	Sporting and music events sponsored by the brand with branded materials (team shirts, banners, etc.) present.	Esports games, leagues, and tournaments sponsored by brands in similar manner to traditional sports.

### Video commercials

Video commercials were the dominant marketing medium for food companies for many years, with many memorable marketing campaigns that have contributed substantially to brand development (27). Commercials use entertainment, emotional, and pop-cultural appeals to capture the attention of the viewer and establish brand identity and equity (28, 29). The brand identity encompasses a set of characteristics or values of the brand that can resonate with a potential consumer or that can provide an aspirational ideal (30). Brand equity is financial benefit derived from consumers’ perception of the brand (31). These adverts have been integrated into many different digital platforms, including streaming services that take the place of television, often with the option given to the viewer to skip the commercial after 5 s.

Television commercials have been the primary exposure media in much food marketing research to date. Multiple systematic reviews have been conducted on the associations between television viewing and food intake and the acute effect of commercial exposure with eating behavior outcomes, such as food choice, preference, and snack intake (1, 3, 32, 33). Consistently, exposure to food brand commercials leads to increased positive attitudes towards food brands and ultimately food choice (3, 34). Particularly in adolescents exposure to commercial advertising has been found to be positively associated with social norms related to the consumption of energy dense foods and sugary beverages (34). The effect of commercials extends beyond the specific brand featured in the commercial and appear to impact intake of general product categories as well (35). The Quantity of food and beverage video commercials targeting children present on television has been associated with the amount of children’s reported intake of fast food and sugar sweetened drinks (36). Additionally, there is compelling evidence that experimental exposure to food commercials leads to an increase in quantity of food intake in children (33, 37). Specifically, food commercials act as a powerful food cue that generally induces a desire to consume or seek out food (24, 38).

### Display advertising

Digital display advertising is most comparable to print advertising, in particular advertisements placed within magazines. In one study, exposure to snack food advertisements present in a children’s magazine were found to result in greater likelihood of choosing the advertised food for a snack (39). Children’s magazines have also been found to contain links to the food and beverage brands websites that encourage further engagement with the brand through giveaways and games (40). Another way that exposure to display advertising has been explored is through looking at outdoor built environments containing food brand billboards (41, 42). The amount of outdoor food and beverage advertising has been associated with greater obesity risk (43).

**Figure 2:**
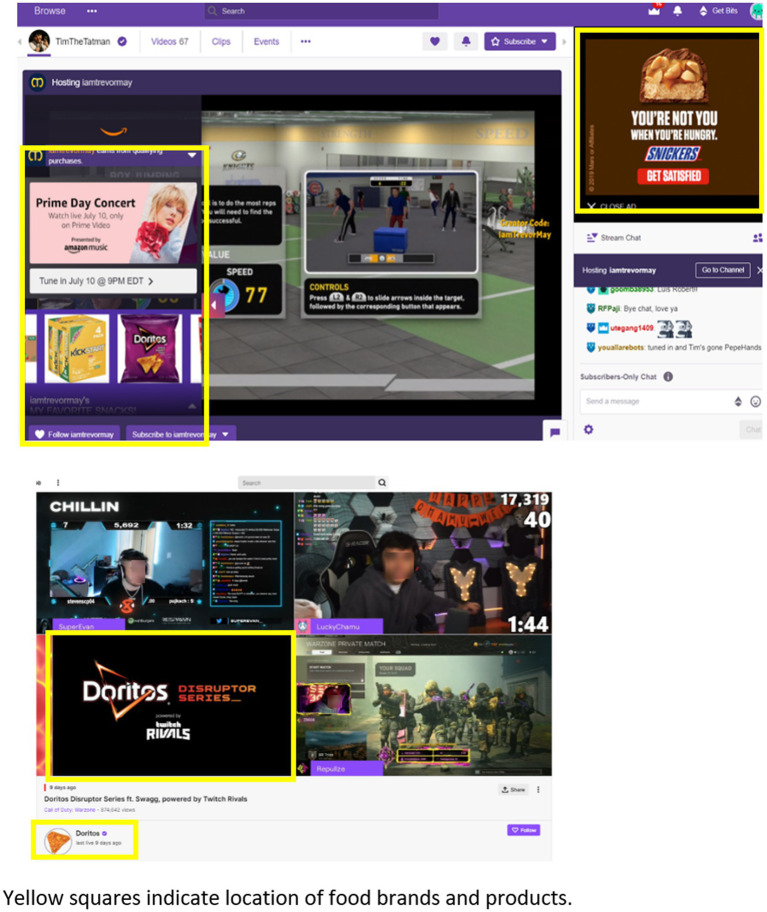
Example of display advertising on Twitch livestream platform. Yellow squares indicate the location of food brands and products.

Generally, the impact of display advertisements online is not as well studied in the food marketing literature. Often, display advertisements are taken out of the context in which they are normally viewed (Figure 2). For example, isolated brand logos images have been used in fMRI research showing brain responses in reward regions. Brain response to food and beverage brand images differs among individuals of different weight status (44) and this brain response correlates with food intake when foods are presented in branded packaging (45). Similarly, display advertisements have been used to measure attentional bias to food cues through eye-tracking study designs. Greater attentional bias toward the advertisements was associated with greater snack food intake (46). Exposure to advertisements showing branded food items has been found to significantly increase brand attachment, which is an emotional connection to the brand that often relates to the identity of the consumer (47, 48). Brand attachment is a strong predictor of purchasing behavior (49). The familiarity of the food products or brand logos and associations with palatability derived from exposure over time may be driving the effect on food choice and intake (Table 2).

### Product placement

**Figure 3:**
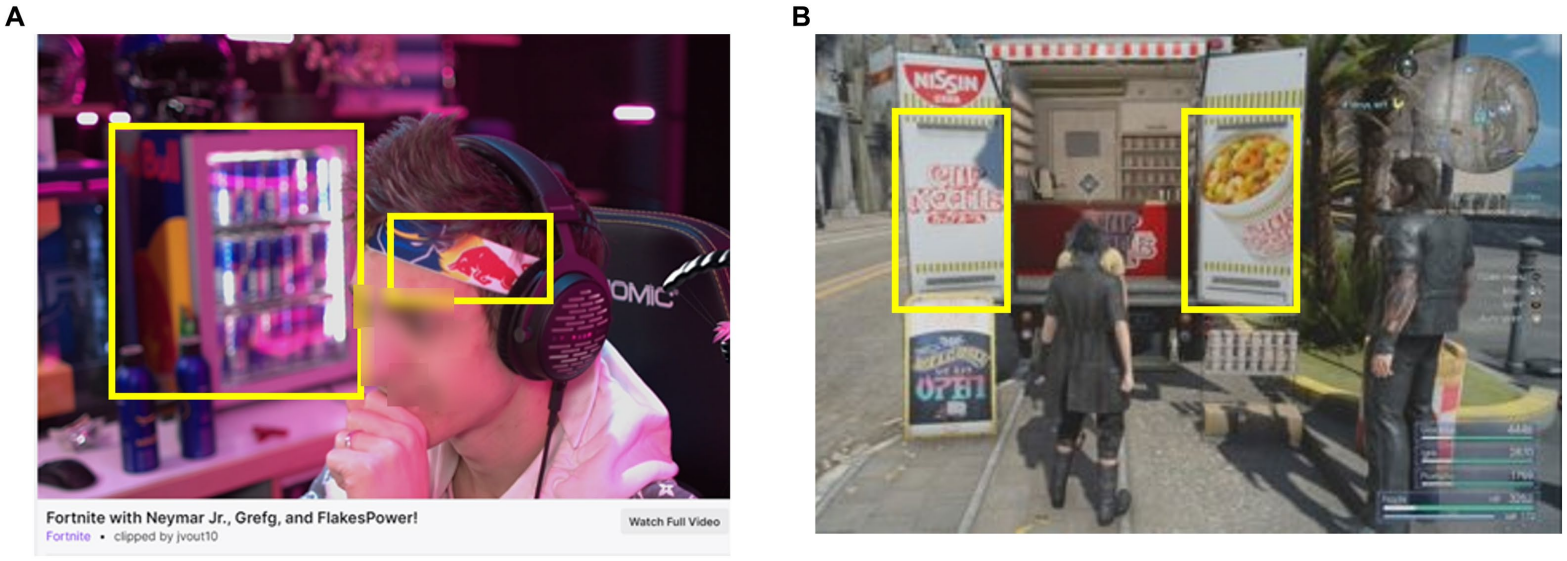
Example of product placement on Twitch livestreaming platform and within a video game. **(A)** Product placement in the streamer’s environment. **(B)** Product placement within a popular video game. Yellow squares indicate location of food brand and product placement.

Product placement is a form of advertising that is integrated directly into the media content with the aim to influence consumer attitudes and behavior toward the featured product (Figure 3) (50, 51). Product placement is an increasingly popular method of advertising currently being leveraged in television, movies, video games, and digital media (52, 53). Brand logos and names can be included as text within digital platforms in the form of content titles and chat discussions. Integration of a product into the entertainment content may reduce consumers’ awareness of the brand placement or the persuasive intent (54, 55). Brand placement in video games has been found to be relatively well received by users and it has been suggested that they may even enhance the realism of certain gaming experiences (56). Product placement influences consumer behavior through repeated exposure, unconscious messaging, and transfer of associations (such as positive emotions) from the entertainment content to the product or brand (57, 58).

The impact of product placement on eating behavior is of considerable interest to researchers (59–61). Exposure to product placement both in television and movies has been shown to influence beverage and food choice in children towards the featured food, independent of brand recall (62, 63). Product placement within digital media has been explored primarily in advergames, which use gamification of brands logos and characters (64). Both branded and unbranded food images have been found to influence food intake in children with a meta-analysis finding a small to moderate effect size (65–67).

### Endorsement

Endorsement of a product by a TV or movie celebrity, musician, and athlete is a historically successful method of marketing used in traditional media, both in video commercials and static adverts (68–70). Food and beverage brands also frequently sponsor events that are linked with endorsements made by the participants of the event and impact children’s attitudes and perferences (70, 71). The mechanism for effective endorsement is centered on meaning transfer and source attractiveness (72, 73). Positive associations of the endorser are transferred to the brand, leading to an increase in brand equity (74). Moreover, the similarity, likability, and familiarity of the endorser to the viewer can enhance the effectiveness of the endorsement (75). In a reciprocal relationship, the traits of the endorser impact brand perceptions and the endorser becomes a part of the brand identity (76). In digital media, specifically social media, endorsement has evolved into influencer marketing, which can be described as a paid partnership between a brand and an influencer (i.e., any person who has a large following on social media) (77). Followers build connection and a sense of familiarity with the influencer that is in reality unknown to that person, defined as a parasocial relationship (78). Influencers use their social leverage and parasocial relationships with followers to increase awareness and positive attitudes toward the brands they promote. Moreover, viewers may regard the sponsorship of content creators as necessary to support content creation and believe that sponsorships are beneficial for both the influencer and the viewer (79).

Online endorsements by both celebrities and influencers are similarly influential on eating behavior (80–82). A systemic review found positive effects of endorsements on beliefs about foods, food choice, and intake in children younger than 12 years (83). Endorsement has been shown to not only increase food intake after exposure to the promotion, but also in subsequent exposure to the endorsing celebrity in a non-food related context (81). Although endorsements are sometimes used for healthier food products, such as the “Got Milk” campaigns, energy dense and nutrient poor foods comprise the majority of celebrity-based endorsements (84, 85).

## Integration of marketing elements in digital media

As our review of the marketing elements attests, prior research provides considerable evidence for the separate impact of video commercials, static adverts, product placement, and endorsement on eating behavior outcomes. However, digital media allows for enhanced integration opportunities using these marketing elements, which creates the potential for saturation, congruency, and social influence to emerge and alter the impact of the advertising effect process. The existing food marketing literature does not conceptualize marketing in the integrative sense and cannot fully account for multi-component strategies (86). However, the concepts of integrated marketing communications and omni-channel marketing, which relate to the coordinated dissemination of brand messaging across a variety of outlets to strategically increase reach and impact and providing an optimized customer experience across platforms, has been explored in the marketing literature (87). This model proposes that integrative effects warrant further explanation and exploration to increase understanding of the impact of digital food marketing on food behaviors. In the following sections, we discuss each aspect of integration in turn, using livestreaming to showcase integration into digital media.

### Saturation

**Figure 4:**
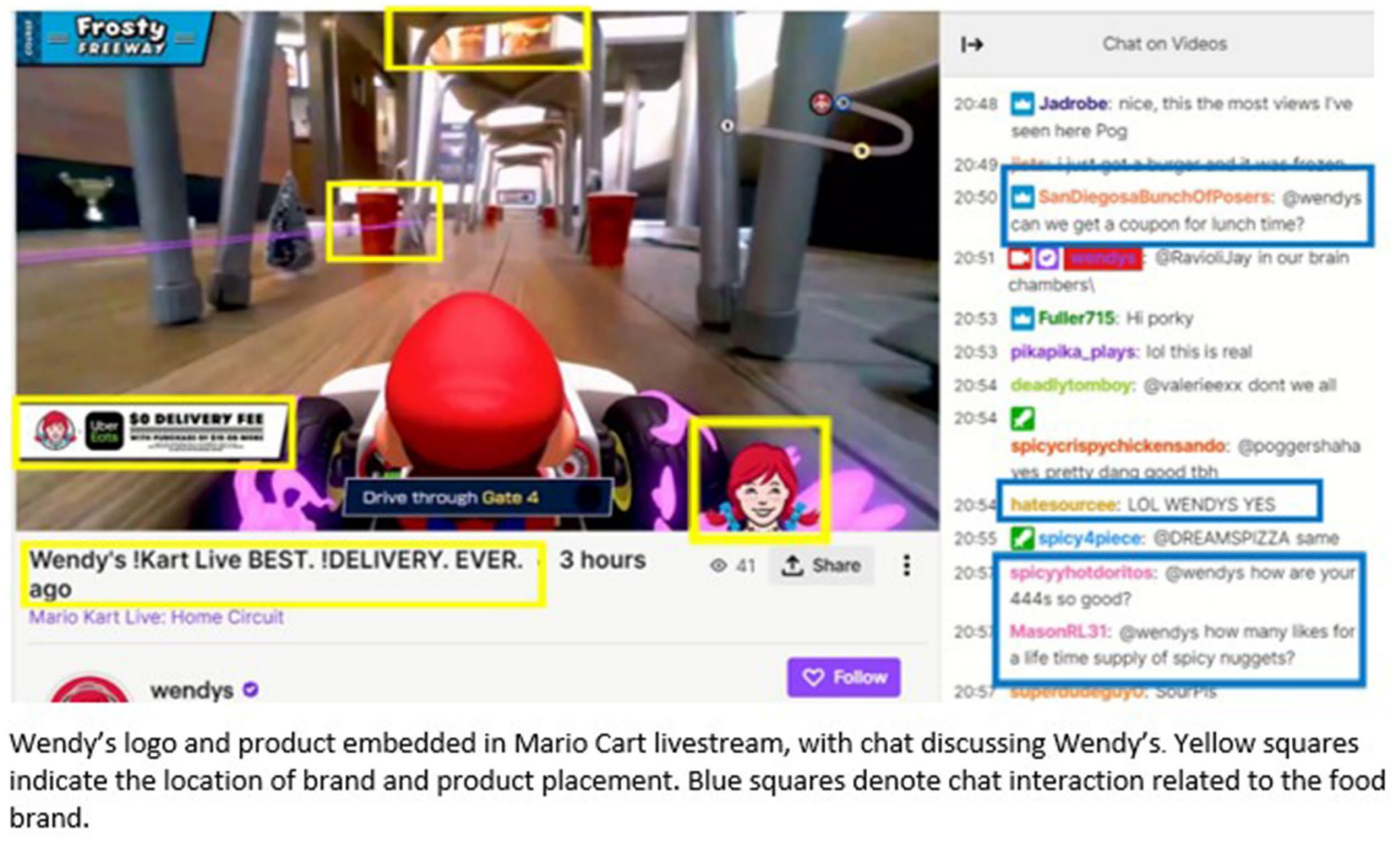
Saturation of marketing on Twitch livestreaming platform. Wendy’s logo and product embedded in Mario Cart livestream, with chat discussing Wendy’s. Yellow squares indicate the location of brand and product placement. Blue squares denote chat interaction related to the food brands.

The possibility of deploying multiple marketing elements in one setting provides an unprecedented way for advertisers to saturate digital media. Unlike traditional media, in digital media multiple marketing elements can be present on the screen simultaneously for extended periods of time, in addition to being embedded within the entertainment content itself. Consider, for example, the livestreaming platform Twitch: streamers appear on screen alongside product placements, with display advertisements layered over the stream and to the side of the broadcast; references to the brand appear in the stream title and in the profile information of the streamer; video commercials are periodically shown during the broadcast; and discussion of the advertised product can occur in the chat space. This is clearly witnessed in a Wendy’s sponsored Mario Cart livestream that created a Wendy’s restaurant Mario Cart course. Here the product and brand logo were heavily integrated into the media content, the video game setting, and the chat discussions (see Figure 4). The chat comments originate both from brand-controlled bots as well as organically from viewers.

**Figure 5:**
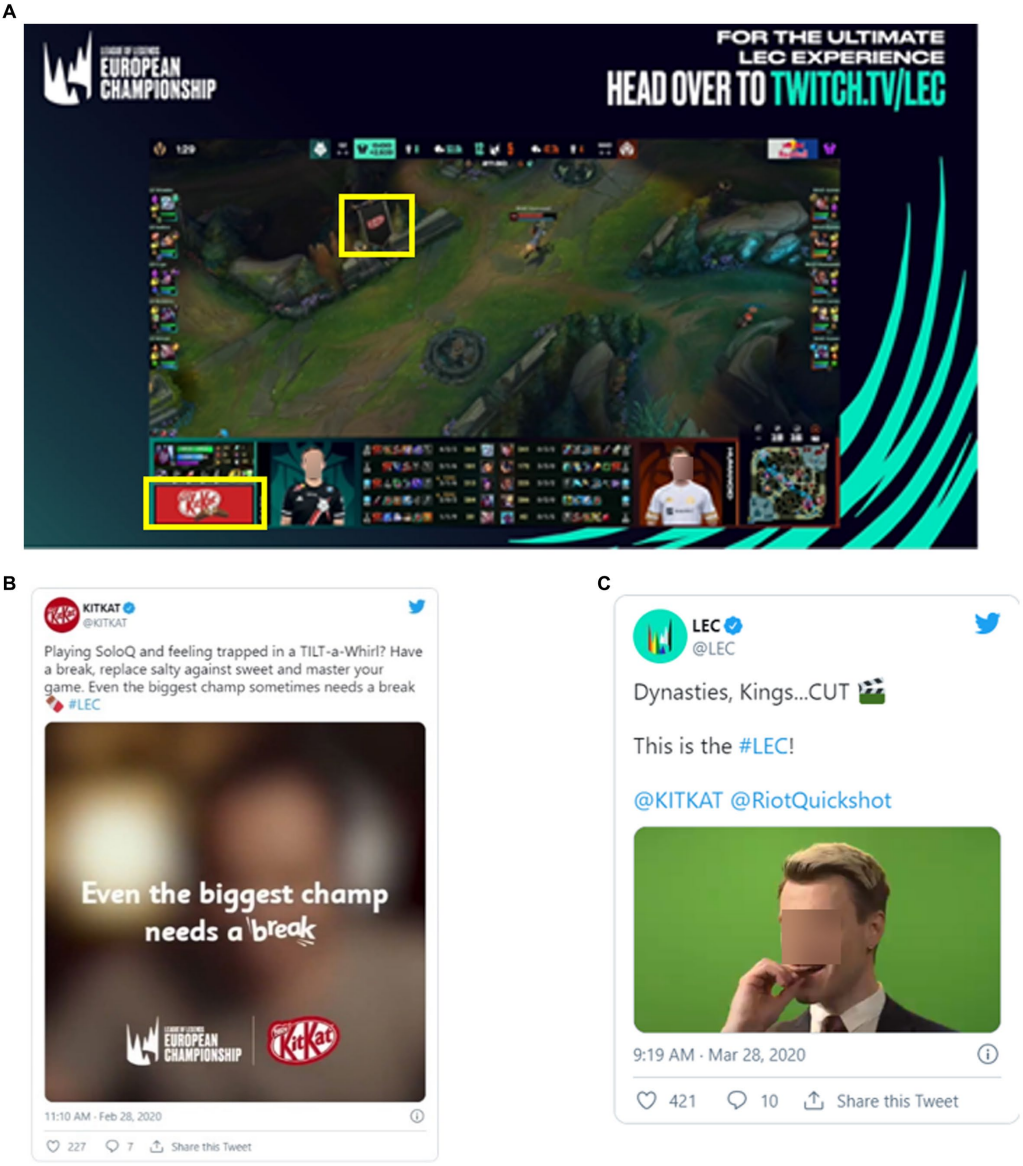
Saturation of marketing across social media (Twitter) and livestreaming platform (Twitch). **(A)** Kit-Kat banner ad within a Twitch livestream and in the video game. **(B)** Kit-Kat twitter page mentioning livestreaming. **(C)** League of legends twitter featuring Kit-Kat. Yellow squares indicate location of embedded brands.

The concept of saturation also extends out beyond the main platform of the marketing campaign. From the perspective of the marketers, this is known as integrated marketing communication and has long been used as a strategy for creating cohesive and synergistic messaging across multiple channels to maximize reach of a marketing campaign (88, 89). For example, Figure 5 depicts layers of static advertising within a League of Legends Tournament with the KitKat logo being integrated into the game itself and into the broadcast content through an overlay advertisement. A simultaneous release of marketing materials was placed onto X, both on KitKat’s company page and the page of the event being broadcast (see Figures 5B,C). In addition, a video commercial campaign was also featured across each of these platforms specifically targeting video gamers (90). Streamers often feature their social media handles during the livestream, which facilitates cross-platform saturation. Digital media presents complications to implementing integrated marketing communications as messaging is harder to control due to user-generated content and input of influencers in brand partnerships (88).

Saturation of marketing with repetitive exposure and multiple points of consumer contact increases the reach and power of the messaging. Prior research specific to multi-element food marketing and eating behavior outcomes is limited, as research has tended to focus on marketing elements in relative isolation. Some preliminary evidence suggests that combining marketing elements can enhance their impact. For example, combining video advertising with product placement in an advergame was shown to increase snack intake over video advertising alone (91). The effects of saturation within a single digital platform has not been explored in the food and beverage marketing literature.

More broadly, saturation may alter the impact of marketing exposure through the level of direct engagement with advertising and in turn the level of conscious processing (92–94). Low to moderate levels of saturation through a variety of marketing elements may lead to an accumulation of incidental contact over time that increases brand recall and nudges viewers to engage with featured brands (95, 96). However, higher levels of saturation could decrease effectiveness through dampened engagement and perhaps an increased perception of intrusiveness (97, 98).

### Congruency

Congruency refers to the degree of likeness or synergy between marketing messages or communications and the entertainment content and is thought to affect the degree of engagement with the marketing content (99). Congruency increases the relevance of the product to the viewer and reduces the perceived intrusiveness of the marketing elements. This is especially important for endorsement-based marketing, as a lack of congruency between the product and the endorser creates distrust (100, 101). Although congruency reduces perceptions of intrusion, incongruent messages increase memory recall (102). The type of media content, viewership, and brands being marketed are important considerations for how congruency impacts attitudes and brand recall (102). Congruency has also been shown to play a role in food marketing, with one study demonstrating that it moderates the impact of advertising on purchasing intent (103).

**Figure 6:**
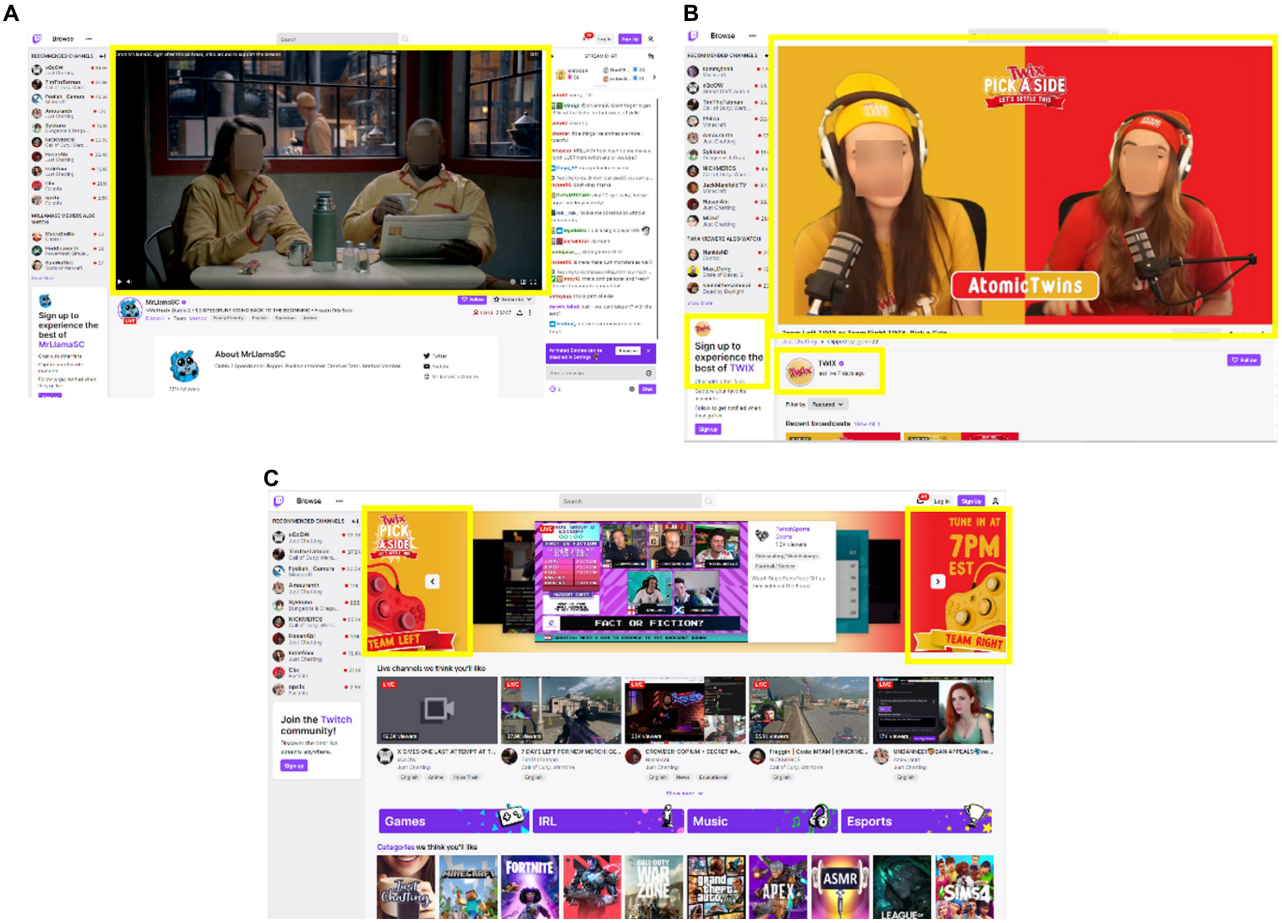
Congruency of marketing and entertainment content on Twitch livestreaming platforms. **(A)** Video commercial before stream starts. **(B)** Brand integration in stream content. **(C)** Brand featured on homepage of Twitch’s website. Yellow squares indicate location of brand logos across the content.

Technology available within digital media allows for multiple strategies for creating congruency between media content and a brand. Lifestyle congruency, which is when the brand relates to shared characteristics of the viewership or those who engage with the specific media the brand is imbedded into, can be achieved with influencer type marketing practices (104). For example, energy drinks are heavily marketed on livestreaming platforms by popular streamers as an important component of their video game playing performance (23). These beverages are integrated into the group group identity of streamers and video game players. Influencers are able to relate a brand to their viewers by describing how they use a product and what they like about it. Livestream video game-based content often utilizes functional congruency, where the brand becomes a component of the game being played. This also creates a virtual direct experience with the product which is more impactful than viewing a branded message alone (105, 106). Congruency may also be achieved by repeated placement of a brand within the entertainment content to enhance the relevance to the viewer. A clear example of this can be seen in the Twix “Pick your side” stream series: a video commercial for Twix plays before streaming content is viewed (Figure 6A), the Twix brand is integrated into a stream (Figure 6B) and featured on the Twitch home screen (Figure 6C). While the Twix candy bar does not directly relate to the video game play itself, elements are included in the overall streaming experience that enhance the congruency of the brand to the media content.

### Social influence

Perhaps one of the most unique and powerful aspects of digital media is the social interaction between content creators and viewers, as well as between other viewers that are perceived as peers. Social influence occurs both from branded content by “influencers” and by viewer generated content. This blurs the line between marketing and entertainment, which further obscures persuasive intent. To bring attention to when content is sponsored by brands, the US Federal Trade Commission as well as the United Kingdom implemented disclosure requirements (such as #ad and #sponsored). These disclosures increase awareness of advertising, but do not always reduce the effectiveness of the marketing messaging (107). The parasocial relationship between the viewer and content creator also likely plays a role in the effectiveness of advertising disclosure (108, 109). Moreover, viewers may regard the sponsorship of content creators as necessary to support content creation and believe that sponsorships are beneficial for both the influencer and the viewer (79).

User-based content, both in reaction to branded content or independently, allows for the development of “brand communities” in which peers share their experiences and knowledge of the brand with other members (110, 111). True to being a community, members develop norms, traditions, values, and specific language (110, 111). Individuals align their behavior, including brand loyalty, to conform to these groups (112). Digital platforms enhance the ability of community members to communicate as well as share user created content, thus strengthening ties to the brand (113, 114). Peers reinforce ideas and behaviors shared within these communities through “liking” and commenting on posts as well as sharing content (115).

**Figure 7:**
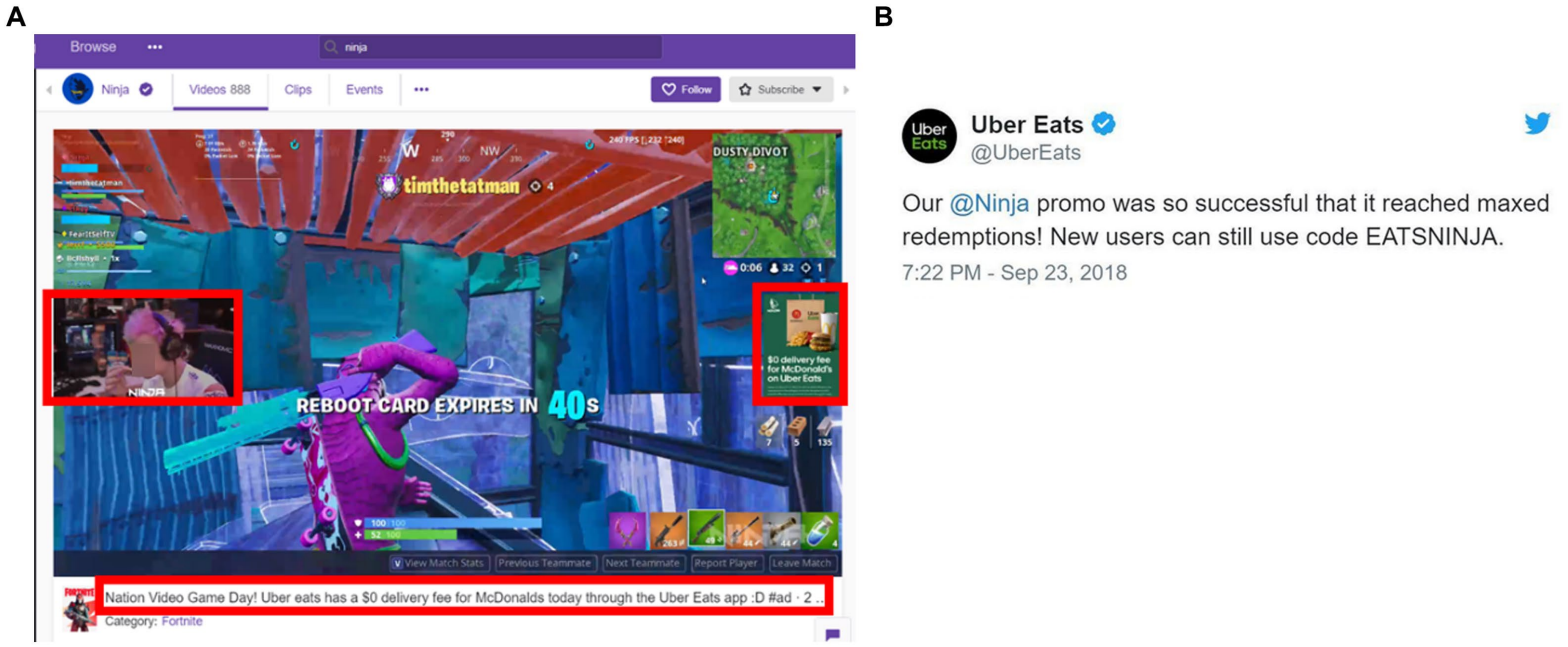
Eating behavioral modeling in Twitch livestreaming platforms. **(A)** Streamer eating McDonald’s with overlay ad and brand mentioned in stream title. **(B)** Uber eats Tweet about campaign. Red squares indicate the location of brands and products.

Both branded and user-generated content can include modeling of eating behaviors. Behavioral modeling may contribute to a transfer of accepted eating behaviors and create new norms within an individual or community (116, 117). The impact of behavioral modeling is likely to be exacerbated on digital media, whose audiences are largely adolescents and young adults and whose attitudes and food behaviors are more susceptible to social influence (79, 118, 119). It is well established that eating behavior can be altered by the influence of others within a given social setting, i.e., social facilitation (116, 120). Social facilitation occurs between both familiar peers and unfamiliar peers (121, 122) and seems likely to emerge on digital platforms by observing influencer behavior. In addition, social facilitation can emerge from perceptions of peer behavior or acceptance of a behavior (123). One example of behavioral modeling and peer reinforcement that occurred on a livestreaming platform involved a streamer partnership with Uber Eats and McDonalds. In addition to the streamer and audience working together to achieve a discount from McDonalds, viewers also observed the streamer ordering the food through Uber Eats, discussing his favorite foods on the menu, and subsequently consuming the food while watching another streamer. In essence, these actions model the behaviors that the food marketing companies would like to reinforce in their customers (see Figures 7A,B).

## Acute exposure outcomes

The FBCDM model categorizes outcome measures to account for the variety of effects food marketing may exert on consumers. This aspect of the model incorporates concepts from the Hierarchy of Unhealthy Food Promotion Effects model (25). The following sections discuss existing digital food marketing research for each outcome type.

### Brand outcomes

Marketing research measures outcomes in terms of brand reach, power, and equity. Important outcome measures that apply to food marketing research with a public health angle are brand awareness (i.e., recall, familiarity), brand attitudes, brand engagement, and purchasing behavior (both intent and actual purchase). The Hierarchy of Unhealthy Food Promotion Effects Model theorizes that these outcomes are important antecedents to consumption behaviors. These outcome alternatives provided valuable insight into the effects of digital marketing that are likely to impact eating behaviors as direct impacts on intake are difficult to measure. Experimental measurement of food intake directly due to digital marketing exposure is challenging as this media is difficult to recreate due to the use of influencers and targeted marketing that individualizes marketing exposures.

For example, recall of food marketing across digital platforms has been associated with self-reported purchase and intake behaviors (124). Greater recall of food marketing may be related to an individual’s attentional bias towards food or individual responsiveness to food cues in the digital environment. In a large sample of livestream users, higher food brand recall was associated with higher external food cue reactivity scores and reported craving of those products (125).

The context in which the food marketing is recalled may impact the attitudes toward the brand, which is important for establishing brand equity (126). Food attitudes have been found to moderate the relationship between brand recall and reported purchase and consumption of these marketed foods (124). Food marketing in digital media has been found to produce more favorable attitudes compared to traditional media (127, 128). The use of digital media has also been associated with higher odds of engagement with multiple food brands, which includes following brands on social media, liking posts or brand pages, or sharing branded content (95). The lasting effect of a digital marketing campaign on positive brand engagement has been demonstrated by examining comments related to a fast-food brand on a popular livestreaming platform (129). Positive commentary increased during the campaign and remained higher than negative comments after the campaign ended. This is also supported by qualitative research in adolescents describing their own perceptions and experiences with food marketing across digital platforms (130, 131). Adolescents in both studies mention the power of frequency, emotional appeal, and degree of relevance to them in shaping their attitudes toward the advertisement and the brand and likelihood of purchasing the products.

### Food outcomes

In comparison to existing food marketing research using television as the exposure media, there are few studies within the digital media space that use measured food or beverage intake in response to acute exposure as the outcome. Most frequently studied are advergames with branded foods imbedded into the game (132). These studies have found a general effect of increased intake, increased intake of energy dense foods compared to lower energy dense foods, and a greater choice of the marketed product compared to an alternative food item (65, 66, 133). Additionally, two studies have examined the effect of social media influencers’ effect on snack intake (80, 107). These studies found exposure to food marketing impacted both amount and type of food consumed. Similarly, a longitudinal study looking at children’s exposure to influencers on video web blogs (vlogs) found that frequency of watching the vlogs related to greater consumption of unhealthy beverages, but not snack food, 2 years later (134).

In place of direct intake measurement, there have been consistent findings using self-reported exposure to digital food marketing and food intake or preferences, specifically in adolescents. In a large sample of adolescents in Belgium, higher exposure to unhealthy food marketing across social media platforms was positively associated with greater intake and preference of those foods (135). Another study that used a narrow definition of digital marketing as receiving a text message or email from a food brand offering a price promotion or giveaway found that the frequency of these messages was associated with greater intake of fast food, sugary drinks, and salty snacks (136). This speaks directly to targeted marketing, which usually results from either location-based data on smart phones, use of food brand apps, or engagement with a brand within digital media. Higher frequency of self-reported engagement with food marketing on social media in the form of liking and sharing content also has been associated with increased intake of unhealthy food and beverages (137).

### Social outcomes

Digital food marketing heavily utilizes social influence, and the effects of exposure may also contribute to perceptions of normative eating behavior and short-term trends. In addition to acute effects on intake, social influence also can contribute to the development of social norms that then contribute to general patterns of behavior (138). Social norm outcomes have primarily been explored using social media platforms Facebook, Instagram, YouTube, Tik Tok, and the adolescent population. Qualitative studies that interviewed adolescents about their perceptions of food marketing within digital media allude to the power of the normative messaging of marketing (130). Key themes included showing food consumed with friend groups who are having fun and who appear happy and depicting food consumed in large quantities. Additionally, a study examining self-reported exposure to food marketing on social media found that greater exposure was associated with higher descriptive norms, or the perceptions of their peer’s behavior, related to consumption of non-core foods (i.e., energy dense, nutrient poor) (135). Social norms related to consuming non-core foods also were found to mediate the relationship between exposure and intake. The effect of exposure to food marketing on social media on intake of non-core foods was in part due to perceptions that peers also consume these foods. In contrast, social norms were not found to mediate the relationship between brand recall of marketed foods within a livestream platform and self-reported intake and purchase behaviors (124).

Exposure to food marketing through the regular use of digital media may have a more general, rather than brand-specific, effect on eating behavior social norms. This may be in part due to the intersection between branded and user generated content that is often difficult to distinguish. The social endorsement of foods and eating behaviors, such as portion sizes, have been shown to influence perceived norms and engagement with marketing content (139–141). A study using Facebook found that adolescents had more positive response to unhealthy food marketing, were more likely to share or like an unhealthy food advertisement or peer post, and rated peers who had more posts of unhealthy than healthy food images more positively (140). Another social platform, Tik Tok, is a source of viral food trends, and adolescents report using this platform to engage with food based content to find new food items to try, watch food preparation techniques, and adopt diet trends (142). Using social based digital content to experimentally examine eating behavior outcomes has resulted in mixed outcomes, likely due to the challenge of recreating the saliency of real peers and influencers (141, 143).

## Long-term impacts

Digital media presents unique challenges for investigating the direct effects on both acute and long-term outcomes (144). The use of data to personalize food and beverage marketing, targeting of specific groups, lack of paid advertising disclosure, and user generated content complication the quantification of exposure and require invasive methods to track long term exposure (14, 145). Emerging evidence shows a link between digital media use and brand awareness, food intake patterns, and social norms (80, 118, 124, 135). However, clear direct effects of marketing exposure and eating behavior outcomes are more difficult to measure. There is a need for more comprehensive and contextual research to establish a clearer picture of the effects of food marketing within digital media to inform policy development. To date purposed bans on both online food and beverage advertising and restrictions on children’s digital data collection for the purpose of selling to markers have not been met with success due to political and industry pushback (146, 147). The FBCDM may be used to develop targeted interventions or policy that balances restriction and impact. For example, the placement or frequency of marketing within a platform, depth of integration into entertainment content, or use of social appeals are potential targets.

Borrowing from the wealth of research on food marketing within traditional media and from what is known about marketing practices within digital media, hypotheses can be developed for potential long-term consequences. Repeated exposure to marketing, combined with positive experiences with the promoted product and brand, is hypothesized to establish brand affinity and loyalty. A loyal consumer base increases the market share for a brand, and this contributes to which products are readily available in consumer markets. Beyond specific brand outcomes, the general categories of food that are marketed are also hypothesized to contribute to overall dietary patterns.

Overwhelmingly high-energy-dense and nutrient-poor foods are marketed across digital platforms. Through the development of social norms around these foods and constant cues for their consumption, habitual intake occurs that may displace healthier food options. Ultimately, health outcomes are therefore hypothesized to be negatively impacted. Digital food marketing exposure may impact both diet quality and energy intake that relates to the risk of chronic disease, such as diabetes and cardiovascular disease, and obesity. Groups at higher risk for chronic disease and obesity due to health inequalities already are disproportionately targeted by food brands (95). Longitudinal data exploring long-term exposure is needed to better quantify the effects on health outcomes in both children and adults.

## Conclusion

Digital media is complex, rapidly evolving, and used extensively for food and beverage marketing. It also affords marketers many new ways to create, combine, and disseminate increasingly persuasive advertising elements and strategies. For example, emerging augmented and virtual reality technology introduces additional immersion and integration of marketing opportunities for food and beverage brands (148, 149). To address the challenges and opportunities presented by this evolving landscape, it is essential to gain an understanding of how food and beverage brands leverage technology within digital media and how viewers are engaging with marketing. The proposed FBCDM model presents a framework to direct research efforts on food and beverage marketing within digital media. The exact relationship and how different combinations of marketing elements and integration strategies contribute to exposure outcomes and long-term impacts warrants further exploration and consideration in future studies. There remains a need for empirical evidence testing of this model and application of the model to other forms of digital media as the primary example explored here was livestreaming. Merging marketing theory with existing food marketing literature can help inform study design to answer these questions more comprehensively. Future studies must carefully consider and account for the variety of manipulations that are possible on digital platforms and the context in which marketing is usually encountered. Another consideration that is important to address in future work would be the impact of individual differences and response to digital marketing as there may be both protective and susceptibility factors. Specifically, the disproportional exposure to unhealthy food and beverages on digital media platforms and parental influence are two areas that warrant further exploration (145, 150). A more comprehensive understanding of digital food and beverage marketing is needed to inform policy that aims to reduce the negative consequences of exposure.

## Data availability statement

The original contributions presented in the study are included in the article, further inquiries can be directed to the corresponding author.

## Author contributions

SM: Conceptualization, Writing – original draft, Writing – review & editing. KK: Writing – review & editing. FD: Writing – review & editing. MV: Writing – review & editing. JF: Writing – review & editing. RE: Writing – review & editing. EB: Writing – review & editing. TM: Conceptualization, Supervision, Writing – review & editing.
